# Effect of dry cow therapy on antimicrobial resistance of mastitis pathogens post-calving

**DOI:** 10.3389/fvets.2023.1132810

**Published:** 2023-07-20

**Authors:** Emmanuel Okello, Wagdy R. ElAshmawy, Deniece R. Williams, Terry W. Lehenbauer, Sharif S. Aly

**Affiliations:** ^1^Veterinary Medicine Teaching and Research Center, School of Veterinary Medicine, University of California, Davis, Tulare, CA, United States; ^2^Department of Population Health and Reproduction, School of Veterinary Medicine, University of California, Davis, Davis, CA, United States; ^3^Department of Internal Medicine and Infectious Diseases, Faculty of Veterinary Medicine, Cairo University, Giza, Egypt

**Keywords:** dry cow therapy, antimicrobial resistance, mastitis pathogens, coagulase negative *Staphylococcus* (CNS), *Streptococcus*, coliforms

## Abstract

The aim of this study was to evaluate the effect of dry cow therapy (DCT) on the antimicrobial resistance (AMR) profile of mastitis pathogens post-calving. A repository of isolates based on a DCT trial was utilized for the current study. A stratified random survey sample of cows from the trial were identified within the strata of season, herd, and trial treatment resulting in 382 cows. All isolates from the 382 cows were selected for the current study, which identified 566 isolates from milk samples collected at dry off (S1), post-calving (S2), and at the first clinical mastitis event up to 150 days in milk (S3). The AMR profiles were determined using broth microdilution method. Less than 10% of the coagulase-negative *Staphylococcus* species (CNS) isolates (*n* = 421) were resistant to tetracycline, ceftiofur, penicillin/novobiocin or erythromycin, while higher proportions of resistance to sulfadimethoxine (72%) and penicillin (28%) were observed. All *Staphylococcus aureus* (*S. aureus)* isolates (*n* = 4) were susceptible to all tested AMD except sulfadimethoxine, to which all isolates were resistant. Similarly, all *Streptococcus* spp. (*n* = 37) were susceptible to penicillin, penicillin/novobiocin, and ampicillin while resistant to tetracycline (17%). All coliforms (*n* = 21) were susceptible to ceftiofur, but resistance was recorded for sulfadimethoxine (70%), cephalothin (56%), and tetracycline (43%). The increased resistance percent from S1 to S2 was observed in CNS isolates from AMD-treated cows, with the highest increase recorded for penicillin (12.2%). Parametric survival interval regression models were used to explore the association between antimicrobial drug (AMD) therapy at dry off and the AMR phenotype post-calving. The accelerated failure-time metric was adopted to minimum inhibitory concentration measurements to permit interpretation of model exponentiated coefficients. Models for cows with CNS isolated at both S1 and S2 showed increased resistance against cephalothin, oxacillin, and ceftiofur in cows that received DCT from the same drug class, or a class with a shared resistance mechanism. In contrast, resistance of CNS isolates to tetracycline were associated with any AMD therapy at dry off. Resistance of CNS isolates to Penicillin decreased in CNS isolates in cows that received any AMD therapy at dry off compared to those that didn't. The study provided evidence that dry-cow IMM AMD was associated with AMR post-calving.

## 1. Introduction

Mastitis is the most economically important disease of dairy cows and a major indication for antimicrobial drug (AMD) use on dairies (1). A recent USDA survey showed that clinical mastitis was detected in approximately one-fourth (24.8%) of all cows at some point in 2013, and cases of clinical mastitis were reported in almost all US dairy operations (99.7%) (2). The same report showed that intramammary antimicrobials were routinely administered to the majority of US dairy cows (89.9%) at dry off (2).

Antimicrobial therapy is a key component of mastitis control programs, commonly administered as an intramammary antimicrobial infusion (IMM) to treat clinical mastitis during the lactation (3), or administered at dry-off to treat existing subclinical infections and prevent new infections during the dry period and early post-partum period (4). At dry-off, intramammary antimicrobials are either administered to all cows (blanket dry cow therapy-BDCT) or selectively to cows at high risk for mastitis during the dry period and early post-partum period (selective dry cow therapy-SDCT). The latter approach is considered a judicious AMD use practice since AMD administration is limited to cows with elevated risk for mastitis that would more likely benefit from such treatment, such as cows with a history of clinical mastitis during the current lactation and cows with high milk somatic cell counts (SCC), which is an indication of subclinical intramammary infection (5).

In the US, a retrospective analysis of 8,905 bacterial isolates obtained from milk samples submitted to the Wisconsin Veterinary Diagnostic Laboratory between 1994 and 2001 showed no specific trend of resistance across drugs over time. For instance, the percentage of *Staphylococcus aureus* (*S. aureus)* isolates resistant to penicillin decreased from 49 to 30%, while percentage of Coagulase negative staphylococci (CNS) isolates resistant to pirlimycin increased from 6 to 19% over the study period which may be due to changes in underlying populations (6). In Canada, a study on resistance profiles of mastitis pathogens on Canadian dairy farms estimated low levels of resistance ranging from 0% (cephalothin and oxacillin) to 8.8% (penicillin) in *S. aureus* isolates, while the estimates for AMR in *Escherichia coli* (*E. coli)* ranged from 0% (ceftriaxone and ciprofloxacin) to 14.8% (tetracycline) (7). Similarly, a study conducted on 934 bacterial isolates from nine European countries during 2009–2012 showed varying levels of resistance to commonly used AMD with 1% resistance against ceftiofur (*S. aureus* and *E. coli*), 14.5, 5.2, and 36.7% resistance against tetracycline for *E. coli, S. aureus*, and *Streptococcus uberis*, respectively, and 25.0% resistance against Penicillin G in *S. aureus* (8).

Most of the previously mentioned studies utilized a cross-sectional study design with no specific information on the AMD exposures of the study cows (7–10). The current study objectives were (1) To utilize a longitudinal study design to characterize the changes in the AMR profiles of bacterial isolates from milk samples collected at dry off, post-calving, and the first mastitis event within 150 days in milk (DIM). (2) To assess the effect of dry cow therapy on antimicrobial resistance of mastitis pathogens in the subsequent lactation. Monitoring the AMD exposure and AMR profile of mastitis pathogens is vital in guiding management strategies to reduce AMD use and minimize the AMD resistance while protecting food safety, animal, and public health.

## 2. Materials and methods

### 2.1. Study design and sampling procedures

**Figure 1:**
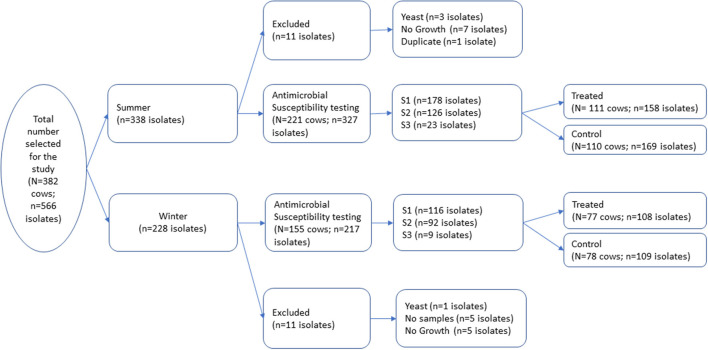
A total of 566 milk pathogen isolates representing 382 cows were selected from a repository of isolates generated from a block randomized field trial for dry cow therapy. Isolates were collected at dry off (S1), post-calving (S2), and at first clinical mastitis prior to 150 days in milk (S3).

Bacterial isolates utilized in this longitudinal study were selected from a repository generated during a randomized blocked field trial conducted on eight California dairies between December 2016 to April 2018 (11). The original trial was approved by the University of California Davis Institutional Animal Care and Use Committee (protocol number 19761). A total of 1,106 cows were enrolled at dry-off on the eight dairies in two seasonal cohorts, fall/winter and spring/summer, and followed to 150 DIM. The study herds were distributed across Northern San Joaquin Valley (NSJV) and Greater Southern California (GSCA) (12). The original trial was conducted to estimate the effect of different dry cow treatments: (1) IMM antimicrobial infusion (AB); (2) Internal Teat Sealant (ITS); (3) Both AB and ITS (AB+ITS); and (4) no treatment (None) on health and production outcomes during the next lactation. The outcome variables evaluated for each treatment group included udder health, milk production and culling during the subsequent lactation. A stratified random survey sample was used to select 382 cows from the trial's 1,106 cows with proportional allocation across the strata season, herd, and treatment. A total of 566 bacterial isolates from milk samples of the 382 cows were utilized for the current study had comparable season, herd, and treatment distribution to the entire repository (Figure 1). Among the selected cows, 192 received IMM AMD infusion (AB or AB+ITS treatment groups) while 190 did not receive IMM AMD infusion and served as controls (ITS or none groups). Sampling stage represented the three timepoints when the milk samples were collected: at dry off and before treatment (S1), post-calving (S2), and at first mastitis event within the first 150 DIM (S3). Intramammary antimicrobial drug infusions used in the study were FDA-approved, commercially available products which included cloxacillin benzathine (Dryclox^®^, Boehringer Ingelheim) (45 cows), ceftiofur hydrochloride (Spectramast DC^®^, Zoetis) (16 cows), cephapirin benzathine (ToMORROW^®^, Boehringer Ingelheim) (85 cows) and a proprietary combination of procaine penicillin G and dihydrostreptomycin (Quartermaster^®^, WG Critical Care, LLC) (46 cows).

### 2.2. Bacterial culture and identification

Bacterial culture and identification were performed following standard protocols used by the National Mastitis Council at the Milk Quality Lab (MQL) at the UC Davis Veterinary Medicine Teaching and Research in Tulare, California (13). Briefly, milk samples were plated on bovine blood agar using calibrated sterile loops and incubated for 24 to 48 h at 37°C. Colony types were identified by colony morphology, hemolysis properties, Gram stain, and biochemical tests. *Staphylococcus aureus* was confirmed by a positive coagulase test; all coagulase-negative *Staphylococcus* species (CNS) isolates were reported as *Staphylococcus* spp. *Streptococcus* spp. were identified by a negative catalase test and *S. agalactiae* was identified by a positive CAMP test and a negative Esculin test. Gram-negative, KOH-positive bacteria were reported as coliforms. All isolates, except *Staphylococcus* spp., were identified to species level by partial sequencing and analysis of 16S RNA gene using 27f and 1492r primers pair as previously described (14).

### 2.3. Antimicrobial susceptibility testing

Antimicrobial susceptibility of the selected isolates was determined by estimating the minimum inhibitory concentration (MIC) using a commercial antimicrobial susceptibility test (AST) plate specific for mastitis pathogens (CMV1AMAF^®^ Sensititre^®^, Thermofisher) and following the manufacturer's procedure. Briefly, 1–5 fresh overnight (24 h) colonies of the bacterial isolate on blood agar (BA) media were resuspended in 5 ml of demineralized water [or 5 ml Mueller-Hinton broth (MHB) for *Streptococcus* spp.] and the concentration adjusted to 0.5 McFarland standard. Next, 10 μl (30 μl for *Streptococcus* spp.) of the bacterial solution was added to 11 ml MHB (or MHB with hemolyzed horse blood for *Streptococcus* spp.) and mixed by repeated inversion of the tube. Fifty microliters of inoculated MHB media were added into each well of the 96-well CMV1AMAF^®^ plate and incubated at 37°C for 18–24 h. The purity and bacterial count in the inoculated MHB broth was checked by taking 1 μl inoculum sample from a positive control well in the AST, streaked on BA, and incubated at 37°C for 18–24 h. The AST plates that had contamination or no growth on corresponding BA were not read, and the test was repeated. The MIC values were read using Sensititre™ Vizion™ Digital MIC Viewing System. The MIC values were recorded as the lowest concentration of antimicrobial drug that inhibited the growth of bacteria. The CMV1AMAF^®^ AST plate contained 10 antimicrobial drugs: ampicillin, penicillin, erythromycin, oxacillin, pirlimycin, penicillin/novobiocin, tetracycline, cephalothin, ceftiofur, and sulfadimethoxine. Susceptibility of the tested isolates against different antimicrobial agents was determined based on CLSI breakpoints (CLSI 2019; VET08, 4th ed). For AMD that did not have established clinical breakpoints the distribution of the MIC values were reported.

### 2.4. Data analyses

The distribution of bacterial isolates by species and seasonal cohort, and susceptibility of the different species against AMD tested were summarized as percentages. The distribution (number) of isolates by MIC interval for each of the drugs in the mastitis AST plate (CMV1AMAF) were summarized by season. Statistical analyses were performed using Stata software (Stata Corp. 2017. Stata Statistical Software: Release 15. College Station, TX: Stata Corp LLC).

### 2.5. Modeling the effect of treatment on MIC values

Models were limited to cows with the same bacterial species isolated at S1 and S2 (*n* = 90 cows). Each model explored the association between AMD therapy at dry off and the AMR phenotype for the drugs available on the CMV1AMAF AST plate. Isolate resistance to a specific drug was measured as a range of MIC values with the lower or upper limits censored (left or right censored, respectively). The original trial treatment groups were explored as an explanatory variable in the model, specifically, AB, ITS, AB+ITS vs. None. Alternative specifications of the treatment variable were explored including a dichotomy comparing AMD therapies (AB or AB+ITS) vs. no AMD therapies at dry off (ITS or None); and based on the type of AMD administered at dry off comparing exposure to, vs. lack of exposure to the dry cow AMD, namely, penicillin (penicillin–dihydrostreptomycin), cephalosporins (ceftiofur hydrochloride or cephapirin benzathine), or cloxacillin benzathine (a semisynthetic beta-lactamase resistant penicillin). Other explanatory variables explored included herd, parity, region, seasonal cohort, breed(s), most recent and highest SCC based on the 6 monthly test records prior to trial enrollment (at dry off), and history of mastitis in the enrollment lactation. In addition, specific observations at enrollment were explored including; California Mastitis Test (CMT) score (negative, trace, 1, 2 or 3) (15); teat end score (1–4; 1 = normal and 4 = very rough cracked teat end with ring); and udder hygiene score (1–4; 1 = clean and 4 = dirty) (16). Finally, the antimicrobial resistance phenotype at S1, and the length of the period between S1 and S2 sampling dates (days) were explored as model covariates.

Interval regression models assume censoring occurs from a normally distributed outcome which may not be true for MIC results. Alternatively, parametric survival interval regression models can be used to model the association between AMD therapy at dry off and the AMR phenotype post-calving. For each drug, AMR was modeled using the exponential, Weibull, Gompertz, lognormal, loglogistic, or generalized gamma distributions. The best fitting parametric distribution was selected based on the lowest Akaike Information Criterion (AIC) estimate for the respective parametric distribution intercept only model. In addition, in the case of the Weibull distribution, its shape parameter (p) and its statistical significance test (H_0_: *p* = 1) was used to confirm whether an exponential or a Weibull distribution was better fitting. Specifically, Weibull distribution was selected over the exponential if the null hypothesis was rejected since the Weibull distribution is reduced to exponential when *p* = 1 (17).

For all models, the accelerated failure-time (AFT) parametrization (instead of hazard) was implemented. The susceptibility of an isolate, measured in MIC was modeled for each drug with robust estimates for standard errors to account for clustering of observations by dairy. To permit interpretation of the model exponentiated coefficients, we introduce the novel nomenclature of an MIC ratio. Identical to AFT model exponentiated coefficients presented as time ratios, the MIC ratio is the quotient resulting from dividing the MIC estimate for a specific covariate profile that represents the exposed (numerator), by that of the unexposed (denominator). As such, an MIC ratio varies from 0 to infinity with 1 indicating no difference between the exposed and unexposed, < 1 indicating that the exposure is protective, or > 1 indicating the exposure is a risk factor. To aid in interpretation, the model predicted MIC were estimated for isolates from cows by dry off treatment status.

Once the best parametric distribution was identified for resistance against each of the study panel's drugs, additional univariate models were specified before the final models were determined using a manual forward building approach. The best fitting model had the lowest AIC value (18) and produced estimates that were within the maximum possible MIC drug concentration (< 1,000,000 μg/ml). Confounding was assessed using the method of change in estimates and biologically plausible interactions determined using statistical significance testing (19).

## 3. Results

### 3.1. Bacterial isolates

A total of 566 isolates were initially selected for the antimicrobial susceptibility testing, but 22 isolates were excluded due to contamination, no growth, missing sample, or duplicate samples as shown in Figure 1. The remaining 544 isolates that were tested for antimicrobial susceptibility are summarized in Table 1. The most common isolates were CNS (*n* = 421), *Streptococcus* spp. (*n* = 37) and *E. coli* (*n* = 19). No *Streptococcus agalactiae* or *Mycoplasma* spp. were isolated from any of the samples. The 16S RNA gene sequences of the isolates were submitted to the GenBank (Accession: OR142768-OR142978).

**Table 1:** Distribution of stratified random sample of milk bacterial isolates of dairy cows selected for antimicrobial susceptibility testing.

**Organism type**	**Season**		**Sampling stage** ^a^			**Treatment group**		**Total**
	**Winter**	**Summer**	**S1**	**S2**	**S3**	**Treated**	**Control**	
*Staphylococcus aureus*	1	3	1	2	1	3	1	4
*Coagulase negative Staphylococcus*	176	245	241	161	19	210	211	421
*Streptococcus* spp.	10	27	13	19	5	15	22	37
*Aerococcus* spp.	2	6	4	3	1	6	2	8
*Lactococcus* spp.	2	4	5	1	0	3	3	6
*Enterococcus* spp.	1	2	2	0	1	2	1	3
*Escherichia coli*	5	14	6	12	1	6	13	19
*Klebsiella*	0	2	0	0	2	0	2	2
*Corynebacterium* spp.	4	13	10	7	0	4	13	17
*Trueperella* spp.	2	1	0	3	3	1	2	3
*Bacillus* spp.	13	10	11	10	2	15	8	23
*Paenobacillus*	1	0	1	0	0	1	0	1
Total	217	327	294	218	32	266	278	544

The selected isolates were stratified by season and sampling stage.

a Sampling stages included dry off (S1), post-calving (S2) and first mastitis event (S3).

### 3.2. Antimicrobial susceptibility

Table 2 summarizes the percent susceptibility of the study isolates to the 10 AMD tested. The four *S. aureus* isolates were susceptible to all AMD tested except sulfadimethoxine, to which all isolates were resistant. More than 90% of the CNS isolates, the most common of all isolates, were susceptible to tetracycline, ceftiofur, penicillin/novobiocin, pirlimycin, and erythromycin. The lowest susceptibility estimate for CNS isolates was for sulfadimethoxine (28%) followed by susceptibility to penicillin (72%). All the *Streptococcus* spp. isolates were susceptible to ampicillin, penicillin and penicillin/novobiocin with more than 90% of the isolates susceptible to erythromycin, pirlimycin, and ceftiofur. In contrast, 17% of *Streptococcus* spp. isolates were resistant to tetracycline. All coliforms (*E. coli* and *Klebsiella* spp.) isolates were susceptible to ceftiofur, while 43% were resistant to tetracycline and 56% were resistant to cephalothin. The lowest susceptibility for coliforms was recorded against sulfadimethoxine (30%).

**Table 2:** Antimicrobial susceptibility of mastitis bacterial isolates cultured from milk samples of dairy cows.

**Organism type**	**N**	**Percent susceptibility against select antimicrobial drugs** ^*^									
		**AMP**	**PEN**	**ERY**	**OXA**	**PIRL**	**P/N**	**TET**	**CEP**	**XNL**	**SDM**
*Staphyococcus aureus*	4		100	100	100	100	100	100	100	100	0
*Staphylococcus spp*. (CNS)	421		72	94		95	98	95		99	28
*Streptococcus spp*.	37	100	100	94		97	100	83		93	
Coliforms (*E. coli* and *Klebsiella*)	21							57	44	100	30
*Corynebacterium spp*.	17	88	71					76	100	76	

Cows were enrolled over winter and summer seasons with milk samples collected from enrolled cows at dry off, post-calving and the first mastitis event within 150 days in milk.

* Ampicillin (AMP), penicillin (PEN), erythromycin (ERY), oxacillin (OXA), pirlimycin (PIRL), penicillin/novobiocin (P/N), tetracycline (TET), cephalothin (CEP), ceftiofur (XNL) and sulfadimethoxine (SDM). Grayed cells represent species drug combinations without MIC breakpoints.

The distribution of MIC values for the CNS isolates, stratified by season, are summarized in Tables 3, 4. The distribution of MIC values for the CNS isolates, stratified by treatment, are summarized in Appendix Tables 1.1, 1.2. In addition, Appendix Tables 1.3–1.10 summarize the MIC distribution of *Staphylococcus aureus, Staphylococcus* spp. (CNS), *Streptococcus* spp., and *Escherichia coli*, stratified by season.

**Table 3:** Percent of susceptible isolates (*n* = 176) by minimum inhibitory concentration (MIC; μg/ml) for *Staphylococcus* spp. (CNS) isolated from milk samples collected from dairy cows during fall/winter season.

	**Percent** ***Staphylococcus*** spp. isolates inhibited at different drug concentrations^*^													**MIC 50**	**MIC 90**
Drug concentration (μg/ml)	≤ 0.12	0.25	0.5	1	2	4	8	16	32	64	128	256	>256		
Ampicillin	74	10	5	2	2	3	3	1						≤ 0.12	2
Penicillin	73	**9**	3	3	0	2	3	7						≤ 0.12	16
Erythromycin		41	46	3	1	1	**9**							1	1
Oxacillin					95	1	4							2	2
Pirlimycin			87	9	1	**1**	3							1	1
Penicillin/Novobiocin				98	0	**1**	0	2						1	1
Tetracycline				89	5	1	1	4						1	1
Cephalothin					95	2	1	0	2					2	2
Ceftiofur			60	31	7	1	**2**							1	2
Sulfadimethoxine									25	1	1	1	**73**	>256	>256

Milk samples were collected from enrolled cows at dry off, post-calving and the first mastitis event within 150 days in milk.

* Bold and underlined estimates signify isolate frequency resistant at the MIC cutoff for the respective drugs (CLSI 2019; VET08, 4th ed). Gray cells represent absence of the respective drug concentration on the plate (CMV1AMAF^®^ Sensititre^®^, Thermofisher).

**Table 4:** Percent of susceptible isolates (*n* = 245) by minimum inhibitory concentration (MIC; μg/ml) for *Staphylococcus* spp. (CNS) isolated from milk samples collected from dairy cows during spring/summer season.

	**Percent** ***Staphylococcus*** spp. isolates inhibited at different drug concentrations^*^													**MIC 50**	**MIC 90**
Drug concentration (μg/ml)	≤ 0.12	0.25	0.5	1	2	4	8	16	32	64	128	256	>256		
Ampicillin	73	9	3	3	3	1	2	5						≤ 0.12	2
Penicillin	72	**9**	2	2	1	1	2	9						≤ 0.12	16
Erythromycin		33	57	4	1	2	**4**							1	1
Oxacillin					96	1	3							2	2
Pirlimycin			87	6	3	**1**	4							1	1
Penicillin/Novobiocin				98	0	**0**	1	1						1	1
Tetracycline				93	2	0	0	4						1	1
Cephalothin					96	0	1	1	1					2	2
Ceftiofur			43	44	10	1	**1**							1	2
Sulfadimethoxine									27	1	2	0	**71**	>256	>256

Milk samples were collected from enrolled cows at dry off, post-calving and the first mastitis event within 150 days in milk.

* Bold and underlined estimates signify isolate frequency resistant at the MIC cutoff for the respective drugs (CLSI 2019; VET08, 4th ed). Gray cells represent absence of the respective drug concentration on the plate (CMV1AMAF^®^ Sensititre^®^, Thermofisher).

### 3.3. Changes in resistance of isolates between dry off and post-calving

Table 5 compares the AMR patterns in CNS isolates at dry off (S1) and post-calving (S2) for cows that did or did not receive IMM AMD at dry off. Estimates for change in resistance of CNS against ampicillin, oxacillin and cephalothin were not assessed due to undefined CLSI MIC breakpoints (CLSI 2019; VET08, 4th ed). The greatest net difference in antimicrobial resistance (DAMR) against the tested AMDs, for CNS strains isolated from treated cows (AB or AB+ITS) between dry off and post-calving, showed a 12.2% increase in DAMR against penicillin, and a single negative DAMR for resistance to tetracycline (−4.9%). In contrast, the DAMR between S1 and S2 samples for CNS isolated from non-treated cows (ITS or control) for the same drugs, showed a greater increase in DAMR for penicillin (16.4%) and no change for tetracycline (0%). On the other hand, in addition to the 16.4% DAMR for penicillin in non-treated cows being the most increase, the only negative DAMR were for resistance to sulfadimethoxine (−6.1%) and ceftiofur (−2.0%).

**Table 5:** Effect of antimicrobial therapy on change in the resistance of Coagulase Negative *Staphylococcus* (CNS) isolated at dry off and post-calving.

**Anti-microbial agents**	**No Antimicrobial therapy at dry off**							**Antimicrobial therapy at dry off**						
	**S1 (***N*^a^ = **49)**			**S2 (***N*^a^ = **49)**			**DAMR**	**S1 (***N*^a^ = **41)**			**S2 (***N*^a^ = **41)**			**DAMR**
	***n***	**%**	**95% CI**	***n***	**%**	**95% CI**	**(%)**	***n***	**%**	**95% CI**	***n***	**%**	**95% CI**	**(%)**
Penicillin	13	26.5	(15.96–40.72)	21	42.9	(29.71–57.10)	16.4	9	21.9	(11.73–37.31)	14	34.1	(21.23–49.94)	12.2
Erythromycin	3	6.1	(1.96–17.57)	5	10.2	(4.26–22.50)	4.1	0	0.0		3	7.3	(2.34–20.63)	7.3
Pirlimycin	2	4.1	(1.00–15.16)	2	4.1	(1.00–15.16)	0.0	0	0.0		3	7.3	(2.34–20.63)	7.3
Pen/Novo^b^	1	2.0	(0.20–13.43)	1	2.0	(0.20–13.43)	0.0	0	0.0		1	2.4	(0.33–0.15.75)	2.4
Tetracycline	2	4.1	(1.00–15.16)	2	4.1	(1.00–15.16)	0.0	4	9.8	(3.66–23.53)	2	4.9	(1.19–17.80)	−4.9
Ceftiofur	1	2.0	(0.20–13.4)	0	0.0		−2.0	0	0.0		1	2.4	(0.33–0.15.75)	2.4
SDMS^c^	38	77.5	(63.63–87.21)	35	71.4	(57.15–82.41)	−6.1	29	70.7	(54.99–82.70)	32	78.0	(62.69–88.27)	7.3

Difference in antimicrobial resistance (DAMR) for each antimicrobial drug was estimated as the change in percentage of resistant isolates between dry off (S1) and post-calving (S2). The change in resistance was evaluated for cows stratified by exposure to intramammary antibiotics. Antimicrobial drugs without established MIC breakpoints (Ampicillin, Oxacillin and Cephalothin) were excluded.

a N is the total of CNS isolates at each sampling stage; n is the number of isolates resistant to specific antibiotics.

b Penicillin/Novobiocin.

c Sulfadimethoxine.

### 3.4. Parametric survival interval regression models

Parametric survival interval regression models were limited to cows (*n* = 86) with CNS species isolates (*n* = 172) from samples collected at both dry off and post-calving due to the low frequency of the other species isolates (0 to 37 isolates). The Weibull distribution was the best fitting for all study models. Models for AMR against penicillin/novobiocin for cows that had CNS at dry off and post-calving were not specified since CNS isolates from 85 of the 86 cows were susceptible to penicillin/novobiocin at ≤ 1 μg/ml and only a single isolate showed resistance at 8 μg/ml. Similarly, a model for post-calving CNS isolates' resistance against sulfadimethoxine couldn't be specified reliably due to extreme right censoring (22 of 23 isolates resistant or 95.7%) resulting in estimates greater than the logical drug concentration (MIC 10^6^ μg/ml).

Final models for resistance in CNS against different AMD are summarized in Tables 6–8 and their predictions by treatment status are presented in Table 9. Model predictions represent the MIC (μg/ml) estimates for the CNS isolates at S2 for each drug.

**Table 6:** Final parametric survival interval regression models for penicillin, ampicillin, and oxacillin resistance in *Staphylococccus* spp. (Coagulase Negative *Staphylococcus*) isolated post-calving.

**Tested drug**	**Variables**	**Levels**	**Coefficient**	**SE**	***P*-value**	**MIC ratio**	**95% CI**
Penicillin	IMM infusion at dry off^a^	No treatment	Referent				
		Treatment	−3.65	1.792	0.04	0.03	(0.0008, 0.87)
	Region	Southern SJV	Referent				
		Northern SJV	−6.64	3.250	0.04	0.001	(2.23e-06, 0.76)
	Interaction (IMM infusion at dry off X Region)^b^		8.35	3.336	0.01		
	Mastitis during dry off lactation	No	Referent				
		Yes	−3.75	1.899	0.04	0.02	(0.0006, 0.97)
	Udder hygiene score	≤ 2	Referent				
		> 2	−4.79	2.380	0.04	0.008	(7.83e-05, 0.88)
	Teat end score	< 4	Referent				
		4 at any teat	6.95	2.914	0.01	1048.23	(3.47, 316, 591.4)
	Intercept		1.12	1.663	0.50	3.06	(0.12, 79.76)
Ampicillin	IMM infusion at dry off^a^	No treatment	Referent				
		Treatment	−0.73	1.293	0.57	0.48	(0.04, 6.09)
	California mastitis test score at dry off	< 3	Referent				
		= 3	−3.14	1.542	0.04	0.04	(0.002, 0.88)
	Intercept		−2.08	1.664	0.21	0.12	(0.004, 3.26)
Oxacillin	IMM infusion at dry off	No treatment	Referent				
		Treatment with cloxacillin	1.83	0.144	< 0.01	6.21	(4.68, 8.23)
		Treatment other than cloxacillin	0.60	0.434	0.16	1.83	(0.78, 4.28)
	Mastitis during dry off lactation	No	Referent				
		Yes	−3.23	0.065	< 0.01	0.04	(0.03, 0.04)
	Parity	= 2	Referent				
		> 2	3.27	0.062	< 0.01	26.26	(23.25, 29.66)
	California mastitis test score at dry off	< 3	Referent				
		= 3	0.97	0.702	0.16	2.63	(0.66, 10.43)
	Intercept		−4.03	0.182	< 0.01	0.02	(0.01, 0.03)

IMM, Intramammary.

a Any antimicrobial drug (AMD) therapy: cloxacillin benzathine (Dryclox^®^, Boehringer Ingelheim), ceftiofur hydrochloride (Spectramast DC^®^, Zoetis), cephapirin benzathine (ToMORROW^®^, Boehringer Ingelheim), and combination of procaine penicillin G and dihydrostreptomycin (Quartermaster^®^, WG Critical Care, LLC).

b MIC ratio estimate comparing post-calving AMR against penicillin in CNS isolates from cows treated at dry-off vs. those untreated = 110.1 (SE 242.87); 95% CI 0, 586.2; P-value 0.65.

**Table 7:** Final parametric survival interval regression models for pirlimycin, erythromycin and tetracycline resistance in *Staphylococccus* spp. (Coagulase Negative *Staphylococcus*) isolated post-calving.

**Tested drug**	**Variables**	**Levels**	**Coefficient**	**SE**	***P*-value**	**MIC ratio**	**95% CI**
Pirlimycin	IMM infusion at dry off^a^	No treatment	Referent				
		Treatment	0.21	1.354	0.87	1.23	(0.09, 17.50)
	Parity	= 2	Referent				
		> 2	3.35	1.467	0.02	28.43	(1.61, 503.69)
	Breed	Pure breed	Referent				
		Mixed breed	−9.85	2.241	< 0.01	5.29e-05	(3.94e-07, 0.006)
	Mastitis during dry off lactation	No	Referent				
		Yes	−9.76	2.420	< 0.01	5.78e-05	(5.94e-07, 0.004)
	Intercept		−5.53	2.008	< 0.01	0.003	(7.73e-05, 0.20)
Erythromycin	IMM infusion at dry off^a^	No treatment	Referent				
		Treatment	0.009	0.432	0.98	1.01	(0.43, 2.35)
	Intercept		−0.44	0.312	0.16	0.65	(0.35, 1.19)
Tetracycline	IMM infusion at dry off^a^	No treatment	Referent				
		Treatment	3.23	0.399	< 0.01	25.24	(11.55, 55.16)
	Intercept		−7.87	2.617	< 0.01	3.83e-04	(2.27e-06, 0.06)

AMD, Antimicrobial drug; IMM, Intramammary.

a Any antimicrobial drug (AMD) therapy: cloxacillin benzathine (Dryclox^®^, Boehringer Ingelheim), ceftiofur hydrochloride (Spectramast DC^®^, Zoetis), cephapirin benzathine (ToMORROW^®^, Boehringer Ingelheim), and combination of procaine penicillin G and dihydrostreptomycin (Quartermaster^®^, WG Critical Care, LLC).

**Table 8:** Final parametric survival interval regression models for cephalothin and ceftiofur resistance in *Staphylococccus* spp. (Coagulase Negative *Staphylococcus*) isolated post-calving.

**Tested drug**	**Variables**	**Levels**	**Coefficient**	**SE**	***P*-value**	**MIC ratio**	**95% CI**
Ceftiofur	IMM infusion at dry off	No treatment	Referent				
		Treatment^a^	0.29	0.114	0.01	1.33	(1.06, 1.66)
	Season	Winter	Referent				
		Summer	0.37	0.185	0.04	1.44	(1.00, 2.07)
	Breed	Pure breed	Referent				
		Mixed breed	0.34	0.030	< 0.01	1.41	(1.33, 1.50)
	Region	Southern SJV	Referent				
		Northern SJV	−0.31	0.116	< 0.01	0.73	(0.58, 0.92)
	Intercept		−0.58	0.114	< 0.01	0.56	(0.45, 0.70)
Cephalothin	IMM infusion at dry off	No treatment	Referent				
		Treatment with cephalosporins	6.43	0.640	< 0.01	620.83	(177.21, 2,175.04)
		Treatment other than cephalosporin	7.10	1.031	< 0.01	1214.22	(160.91, 9162.45)
	Intercept		−9.35	1.285	< 0.01	8.67e-05	(< 0.0001, 0.001)

IMM, Intramammary.

a Any antimicrobial drug (AMD) therapy: cloxacillin benzathine (Dryclox^®^, Boehringer Ingelheim), ceftiofur hydrochloride (Spectramast DC^®^, Zoetis), cephapirin benzathine (ToMORROW^®^, Boehringer Ingelheim), and combination of procaine penicillin G and dihydrostreptomycin (Quartermaster^®^, WG Critical Care, LLC).

**Table 9:** Parametric survival interval regression model predicted Minimum Inhibitory Concentration (MIC) for *Staphylococccus* spp. (Coagulase Negative *Staphylococcus*) isolated post-calving from dairy cows by dry off antimicrobial drug (AMD) treatment status.

**Model predicting AMR against:**	**Dry off AMD**	**Treated group**		**Non treated group**		**MIC Difference**		***P*-value**
		**Coefficient**	**SE**	**Coefficient**	**SE**	**Estimate**	**SE**	
Penicillin (treatment by region interaction)	Any AMD^a^ (S. SJV)	0.08	0.081	3.06	5.094	−2.98	5.08	0.55
	Any AMD (N. SJV)	0.44	0.248	0.004	0.009	0.44	0.25	0.07
Ampicillin	Any	0.06	0.032	0.12	0.208	−0.06	0.184	0.72
Oxacillin	Cloxacillin	0.11	0.007	0.02	0.003	0.09	0.005	< 0.01
	Treatment other than cloxacillin	0.03	0.013	0.02	0.003	0.015	0.014	0.28
Cephalothin	Cephalosporins	0.05	0.054	< 0.01	< 0.01	0.05	0.054	0.32
	Cephalosporins	0.11	0.048	< 0.01	< 0.01	0.11	0.047	0.02
Ceftiofur	Any	0.74	0.144	0.56	0.063	0.18	0.096	0.05
Pirlamycin	Any	0.005	0.007	0.004	0.007	< 0.01	0.005	0.86
Tetracycline	Any	0.01	0.023	< 0.01	0.001	0.01	0.022	0.67
Erythromycin	Any	0.64	0.286	0.65	0.201	0.01	0.281	0.98

a Any antimicrobial drug (AMD) therapy: cloxacillin benzathine (Dryclox^®^, Boehringer Ingelheim), ceftiofur hydrochloride (Spectramast DC^®^, Zoetis), cephapirin benzathine (ToMORROW^®^, Boehringer Ingelheim), and combination of procaine penicillin G and dihydrostreptomycin (Quartermaster^®^, WG Critical Care, LLC).

Models for resistance of CNS post-calving to oxacillin, tetracycline, cephalothin and ceftiofur showed positive associations with exposure to dry off IMM therapy using AMD from the same drug class, a class with a similar resistance mechanism, or any AMD at dry off. In contrast, models for resistance of CNS to penicillin post-calving identified a negative association with exposure to any AMD at dry off. There were no associations between any dry off IMM AMD therapy and CNS resistance post-calving against the remaining drugs (ampicillin, pirlimycin or erythromycin).

Several cow-related factors were predictive of CNS isolate AMD at S2. Specifically, history of previous mastitis in the dry off lactation was associated with a decrease in resistance to penicillin, oxacillin and pirlimycin post-calving, in comparison to cows with no history of mastitis. In addition, a teat-end score four in any of the four quarters at dry off was associated with increased resistance of CNS to penicillin post-calving. However, CNS isolates from cows with udder hygiene score >2 at dry off had lower resistance to penicillin post-calving compared to cows with cleaner udders (lower hygiene scores). Cows with CMT score of three at dry-off had significantly lower resistance against ampicillin compared to cows with lower scores. Higher parity (>3 lactation) was associated with significant increase in CNS resistance to oxacillin compared to lower parity.

Region was only predictive of resistance of CNS to penicillin and ceftiofur post-calving; isolates from study cows in the NSJV herds showed less resistance than their counterparts in the GSCA herds. Seasonal changes were also observed, where CNS resistance to ceftiofur post-calving was higher in the summer compared to winter. Interestingly, after adjusting to dry off treatment, management, and cow factors, resistance at dry off was not predictive of resistance of CNS isolates post-calving to the same AMD across all models. Model predictions of MIC of CNS isolates at S2, by dry-off treatment status and difference between treated and untreated cows are summarized in Table 9.

## 4. Discussion

Coagulase negative *Staphylococcus* spp. (CNS) was the most common bacterial type isolated from milk samples collected across all the sampling stages and seasons of a previously described dry cow therapy trial in California. Similarly, other recent studies have reported CNS as the most common mastitis isolate in many regions (20–22). *Streptococcus* spp. and coliforms were the second and third most common isolates, respectively, with relatively lower frequencies compared to CNS. Coliforms are mainly associated with clinical environmental mastitis and are thus expected to occur at low frequency in non-clinical cows (23).

Overall, our results showed high susceptibility of CNS isolated from milk to common AMD used for mastitis therapy in dairy cows, in addition to other antimicrobial drugs included in a commercially available mastitis antimicrobial sensitivity testing plate. Similar results were reported in the US (24) and Canadian herds (25). In contrast to high susceptibility of isolates from North American and European countries, study reports from other continents have indicated high prevalence of resistance of mastitis pathogens against commonly used AMD. A recent study from China reported up to 64 and 34% resistance of CNS isolates from large Chinese dairy herds to penicillin and tetracycline, respectively, compared to the corresponding 28 and 5% resistance estimated in this study (26). Similarly, high resistance of CNS and others mastitis pathogens were reported in Ethiopia (27, 28), Jordan (29), and Brazil (30, 31). The finding of high AMR against common mastitis drugs correlates to the general pattern of bacterial resistance in these countries (28, 32, 33).

Despite the low level of resistance reported in this study, the results provide evidence of an association between IMM antimicrobial therapy at dry off and increased resistance of isolates recovered post-calving. Such a finding is an impetus for the development and implementation of stewardship programs that promote judicious use of AMD for mastitis therapy and hence maintain the low resistance status quo. In the US, approximately 93% of dairy cows are treated with AMD at dry off on 80.3% of the dairy herds (2). The current and long standing practice for control of bovine mastitis is to administer IMM antimicrobial infusion in all four quarters of all cows at dry off (blanket dry cow therapy) (34–36). However, recent studies have shown that selective therapy does not have a negative effect on cow health and performance during early lactation when compared to blanket dry cow therapy (11, 37–39). A judicious approach to AMD use would therefore involve identifying only high-risk cows to receive IMM AMD therapy at dry-off, as opposed to blanket therapy. Extension and outreach plans should be implemented to increase the awareness of the stakeholders, including dairy producers and veterinarians, on the development of AMR due to AMD therapy at dry off and strategic approaches for implementation of selective dry cow therapy programs.

The study data showed an association between resistance of post-calving CNS isolates to oxacillin, cephalothin, ceftiofur, and tetracycline, and dry off exposure to AMD from the same drug classes. Resistance associated with AMD therapies from the same drug class could be explained by the fact that bacterial organisms share common mechanisms of resistance to beta-lactam AMD, which include modifications of the drug target, penicillin binding proteins (PBP), or by producing the protective beta-lactamase enzymes. While chromosomal beta-lactamase are species-specific, the plasmid-mediated enzymes are transferrable between bacterial species and genera (40–43).

In contrast, the observed negative association between exposure to AMD at dry off that contained penicillin and penicillin resistance in CNS isolates post-calving. Our finding is in contrast to penicillin administration and resistance to penicillin and ampicillin previously observed in bovine mastitis *Staphylococcus aureus* isolates on Canadian dairy farms (44). The reason for the negative association between penicillin exposure at dry off and reduction in resistance against penicillin in CNS isolates post-calving is not known.

Interestingly, resistance of CNS isolated at dry off was not predictive of resistance post-calving which could be due to sample size. Our results also showed that treatment of cows with any of the AMD used for dry-cow therapy on the study herds resulted in an increase in resistance of CNS isolates to tetracycline post-calving. Most drugs induce selection and/or overexpression of multidrug efflux pumps which contributes to antimicrobial resistance (45). Since drug efflux is a major mechanism of resistance to tetracyclines, any drug that augments this process would potentially cause associated resistance to tetracycline (46–48). In addition, co-resistance to tetracycline and other AMD such as ampicillin, erythromycin, chloramphenicol, streptomycin, neomycin, gentamicin, sulfamethoxazole/trimethoprim was previously reported in staphylococcal isolates from domestic animals (49).

The main limitation of the current study was the small number of Gram positive or Gram negative bacteria isolated from milk samples. Fewer cows had the same species isolated at S1 and S2 making it difficult to compare the effect of the AMD on resistance in the same species. The current study also speciated non Staph isolates. As a result, CNS species were not identified and hence could differ between sampling points. Hence, the observed changes in the MIC values could be due to heterogeneity in AMR associated with different CNS species. In addition, further research is needed to estimate the effect of AMD IMM infusion on the development of AMR in mastitis pathogens other than CNS.

In conclusion, the current study showed low resistance of mastitis pathogens to AMD commonly used for mastitis therapy. However, the study provided evidence that IMM administration of AMD at dry off was associated with an increase in the AMR of CNS isolates post-calving. As such, antimicrobial stewardship on dairies including selection of cows for AMD administration at dry off should be guided by post-calving mastitis risk. Development and validation of a rapid, low cost and effective selective dry-cow therapy algorithm is required on dairy herds to improve antimicrobial stewardship.

## Data availability statement

The datasets presented in this article are not readily available because data collected for this study is protected under California Food and Agriculture Code 14407. Requests for raw data may be made to the authors, who will consult with the California Department of Food and Agriculture (CDFA) and study producers on fulfilling the request. Requests to access the datasets should be directed to SA, saly@ucdavis.edu.

## Author contributions

Conceptualization and funding acquisition: SA, TL, and EO. Methodology and investigation: SA, EO, WE, and DW. Software, supervision, and project administration: SA. Validation and writing original draft preparation: SA, EO, and WE. Formal analysis and data curation: SA and WE. Writing—review and editing: SA, EO, WE, DW, and TL. Visualization: EO. All authors have read and agreed to the published version of the manuscript.
