# Supplementary material for: Effect of dry cow therapy on antimicrobial resistance of mastitis pathogens post-calving
Source: Front Vet Sci. 2023 Jul 20;10:1132810. doi: 10.3389/fvets.2023.1132810 (PMC10399697; doi:10.3389/fvets.2023.1132810)
Supplement: Supplementary file 1 [file Table_1.DOCX]

**Appendix Tables:**

**Appendix 1.1** Percent of susceptible isolates (n=211) by minimum inhibitory concentration (MIC; µg/mL) for *Staphylococcus* spp. (CNS) collected from control group (no intramammary antimicrobials). Milk samples were collected from enrolled cows at dry off, post-calving and the first mastitis event within 150 days in milk.

| Drug concentration | Percent *Staphylococcus* spp. isolates inhibited at different drug concentrations | | | | | | | | | | | | | MIC 50 | MIC 90 |
| --- | --- | --- | --- | --- | --- | --- | --- | --- | --- | --- | --- | --- | --- | --- | --- |
|  | ≤0.12 | 0.25 | 0.5 | 1 | 2 | 4 | 8 | 16 | 32 | 64 | 128 | 256 | >256 |  |  |
| Ampicillin | 74.4 | 10.9 | 4.7 | 2.8 | 1.4 | 1.9 | 1.4 | 2.4 |  |  |  |  |  | ≤0.12 | 0.5 |
| Penicillin | 73.5 | 10.9 | 3.3 | 1.9 | 0.5 | 0.9 | 1.4 | 7.6 |  |  |  |  |  | ≤0.12 | 1 |
| Erythromycin |  | 39.3 | 49.8 | 2.4 | 1.4 | 0.5 | 6.6 |  |  |  |  |  |  | 0.5 | 1 |
| Oxacillin |  |  |  |  | 96.2 | 0.9 | 2.8 |  |  |  |  |  |  | 2 | 2 |
| Pirlimycin |  |  | 88.2 | 5.7 | 1.9 | 0.5 | 3.8 |  |  |  |  |  |  | 0.5 | 1 |
| Penicillin/Novobiocin |  |  |  | 98.1 | 0.0 | 0.5 | 0.0 | 1.4 |  |  |  |  |  | 1 | 1 |
| Tetracycline |  |  |  | 95.7 | 2.4 | 0.5 | 0.0 | 1.4 |  |  |  |  |  | 1 | 1 |
| Cephalothin |  |  |  |  | 97.6 | 0.0 | 0.9 | 0.0 | 1.4 |  |  |  |  | 2 | 2 |
| Ceftiofur |  |  | 53.6 | 37.0 | 8.1 | 0.9 |  |  |  |  |  |  |  | 0.5 | 1 |
| Sulfadimethoxine |  |  |  |  |  |  |  |  | 21.8 | 1.4 | 1.9 | 0.5 | 74.4 | >256 | >256 |

**Appendix 1.2** Percent of susceptible isolates (n=210) by minimum inhibitory concentration (MIC; µg/mL) for *Staphylococcus* spp. (CNS) collected from treatment group (received intramammary antimicrobials). Milk samples were collected from enrolled cows at dry off, post-calving and the first mastitis event within 150 days in milk.

| Drug concentration | Percent *Staphylococcus* spp. isolates inhibited at different drug concentrations | | | | | | | | | | | | | MIC 50 | MIC 90 |
| --- | --- | --- | --- | --- | --- | --- | --- | --- | --- | --- | --- | --- | --- | --- | --- |
|  | ≤0.12 | 0.25 | 0.5 | 1 | 2 | 4 | 8 | 16 | 32 | 64 | 128 | 256 | >256 |  |  |
| Ampicillin | 72.4 | 8.1 | 3.3 | 2.4 | 4.3 | 1.9 | 3.3 | 4.3 |  |  |  |  |  | ≤0.12 | 2 |
| Penicillin | 71.4 | 7.6 | 2.4 | 2.4 | 0.5 | 1.9 | 4.3 | 9.5 |  |  |  |  |  | ≤0.12 | 8 |
| Erythromycin |  | 33.3 | 55.2 | 4.8 | 0.0 | 1.9 | 4.8 |  |  |  |  |  |  | 0.5 | 1 |
| Oxacillin |  |  |  |  | 95.2 | 1.0 | 3.8 |  |  |  |  |  |  | 2 | 2 |
| Pirlimycin |  |  | 85.2 | 8.1 | 1.9 | 1.0 | 3.8 |  |  |  |  |  |  | 0.5 | 1 |
| Penicillin/Novobiocin |  |  |  | 97.1 | 0.5 | 0.5 | 1.0 | 1.0 |  |  |  |  |  | 1 | 1 |
| Tetracycline |  |  |  | 87.6 | 3.3 | 1.0 | 1.4 | 6.7 |  |  |  |  |  | 1 | 2 |
| Cephalothin |  |  |  |  | 94.3 | 1.9 | 1.4 | 1.0 | 1.4 |  |  |  |  | 2 | 2 |
| Ceftiofur |  |  | 46.7 | 40.5 | 9.0 | 1.4 |  |  |  |  |  |  |  | 1 | 2 |
| Sulfadimethoxine |  |  |  |  |  |  |  |  | 30.0 | 0.0 | 0.5 | 0.5 | 69.0 | >256 | >256 |

**Appendix 1.3** Minimum inhibitory concentration (MIC; µg/ml) distribution for *Staphylococcus aureus* in the fall/winter season (n=1)

|  | Percent *Staphylococcus aureus* isolates inhibited at different drug concentrations* | | | | | | | | | | | | | MIC 50 | MIC 90 |
| --- | --- | --- | --- | --- | --- | --- | --- | --- | --- | --- | --- | --- | --- | --- | --- |
| Drug concentration (µg/ml)) | ≤0.12 | 0.25 | 0.5 | 1 | 2 | 4 | 8 | 16 | 32 | 64 | 128 | 256 | >256 |  |  |
| Ampicillin | 100 | 0 | 0 | 0 | 0 | 0 | 0 | 0 |  |  |  |  |  | ≤0.12 | ≤0.12 |
| Penicillin | 100 | **0** | 0 | 0 | 0 | 0 | 0 | 0 |  |  |  |  |  | ≤0.12 | ≤0.12 |
| Erythromycin |  | 0 | 100 | 0 | 0 | 0 | **0** |  |  |  |  |  |  | 1 | 1 |
| Oxacillin |  |  |  |  | 100 | 0 | 0 |  |  |  |  |  |  | 2 | 2 |
| Pirlimycin |  |  | 0 | 100 | 0 | **0** | 0 |  |  |  |  |  |  | 1 | 1 |
| Penicillin/Novobiocin |  |  |  | 100 | 0 | **0** | 0 | 0 |  |  |  |  |  | 1 | 1 |
| Tetracycline |  |  |  | 0 | 0 | 0 | 0 | 100 |  |  |  |  |  | 1 | 1 |
| Cephalothin |  |  |  |  | 100 | 0 | 0 | 0 | 0 |  |  |  |  | 2 | 2 |
| Ceftiofur |  |  | 0 | 100 | 0 | 0 | **0** |  |  |  |  |  |  | 1 | 1 |
| Sulfadimethoxine |  |  |  |  |  |  |  |  | 0 | 0 | 0 | 0 | **100** | >256 | >256 |

* Bold estimates signify isolate frequency resistant at the MIC cutoff for the respective drugs.

**Appendix 1.4** Minimum inhibitory concentration (MIC; µg/ml) distribution for *Staphylococcus aureus* in the spring/summer season (n=3)

|  | Percent *Staphylococcus aureus* isolates inhibited at different drug concentrations* | | | | | | | | | | | | | MIC 50 | MIC 90 |
| --- | --- | --- | --- | --- | --- | --- | --- | --- | --- | --- | --- | --- | --- | --- | --- |
| Drug concentration (µg/ml)) | ≤0.12 | 0.25 | 0.5 | 1 | 2 | 4 | 8 | 16 | 32 | 64 | 128 | 256 | >256 |  |  |
| Ampicillin | 100 | 0 | 0 | 0 | 0 | 0 | 0 | 0 |  |  |  |  |  | ≤0.12 | ≤0.12 |
| Penicillin | 100 | **0** | 0 | 0 | 0 | 0 | 0 | 0 |  |  |  |  |  | ≤0.12 | ≤0.12 |
| Erythromycin |  | 33 | 67 | 0 | 0 | 0 | **0** |  |  |  |  |  |  | 1 | 1 |
| Oxacillin |  |  |  |  | 100 | 0 | 0 |  |  |  |  |  |  | 2 | 2 |
| Pirlimycin |  |  | 100 | 0 | 0 | **0** | 0 |  |  |  |  |  |  | 1 | 1 |
| Penicillin/Novobiocin |  |  |  | 100 | 0 | **0** | 0 | 0 |  |  |  |  |  | 1 | 1 |
| Tetracycline |  |  |  | 100 | 0 | 0 | 0 | 00 |  |  |  |  |  | 1 | 1 |
| Cephalothin |  |  |  |  | 100 | 0 | 0 | 0 | 0 |  |  |  |  | 2 | 2 |
| Ceftiofur |  |  | 0 | 100 | 0 | 0 | **0** |  |  |  |  |  |  | 1 | 1 |
| Sulfadimethoxine |  |  |  |  |  |  |  |  | 100 | 0 | 0 | 0 | **0** | >256 | >256 |

* Bold estimates signify isolate frequency resistant at the MIC cutoff for the respective drugs.

**Appendix 1.5** Minimum inhibitory concentration (MIC; µg/ml) distribution for *Streptococcus* spp in the fall/winter season (n=10)

|  | Percent *Streptococcus* spp. isolates inhibited at different drug concentrations* | | | | | | | | | | | | | MIC 50 | MIC 90 |
| --- | --- | --- | --- | --- | --- | --- | --- | --- | --- | --- | --- | --- | --- | --- | --- |
| Drug concentration (µg/ml)) | ≤0.12 | 0.25 | 0.5 | 1 | 2 | 4 | 8 | 16 | 32 | 64 | 128 | 256 | >256 |  |  |
| Ampicillin | 90 | 10 | 0 | 0 | 0 | 0 | **0** | 0 |  |  |  |  |  | ≤0.12 | 0 |
| Penicillin | 90 | 10 | 0 | 0 | 0 | **0** | **0** | 0 |  |  |  |  |  | ≤0.12 | 0 |
| Erythromycin |  | 80 | 10 | 0 | 10 | 0 | 0 |  |  |  |  |  |  | 0 | 0 |
| Oxacillin |  |  |  |  | 90 | 10 | 0 |  |  |  |  |  |  | 2 | 2 |
| Pirlimycin |  |  | 90 | 10 | 0 | **0** | 0 |  |  |  |  |  |  | 1 | 1 |
| Penicillin/Novobiocin |  |  |  | 100 | 0 | **0** | 0 | 0 |  |  |  |  |  | 1 | 1 |
| Tetracycline |  |  |  | 80 | 10 | 0 | 0 | 10 |  |  |  |  |  | 1 | 16 |
| Cephalothin |  |  |  |  | 90 | 10 | 0 | 0 | 0 |  |  |  |  | 2 | 2 |
| Ceftiofur |  |  | 70 | 0 | 10 | 0 | **20** |  |  |  |  |  |  | 1 | 2 |
| Sulfadimethoxine |  |  |  |  |  |  |  |  | 10 | 0 | 10 | 0 | 80 | >256 | >256 |

* Bold estimates signify isolate frequency resistant at the MIC cutoff for the respective drugs.

**Appendix 1.6** Minimum inhibitory concentration (MIC; µg/ml) distribution for *Streptococcus spp* in the spring/summer season (n=27)

|  | Percent *Streptococcus* spp. isolates inhibited at different drug concentrations* | | | | | | | | | | | | | MIC 50 | MIC 90 |
| --- | --- | --- | --- | --- | --- | --- | --- | --- | --- | --- | --- | --- | --- | --- | --- |
| Drug concentration (µg/ml)) | ≤0.12 | 0.25 | 0.5 | 1 | 2 | 4 | 8 | 16 | 32 | 64 | 128 | 256 | >256 |  |  |
| Ampicillin | 81 | 19 | 0 | 0 | 0 | 0 | **0** | 0 |  |  |  |  |  | ≤0.12 | 0 |
| Penicillin | 89 | 11 | 0 | 0 | 0 | **0** | 0 | 0 |  |  |  |  |  | ≤0.12 | 0 |
| Erythromycin |  | 89 | 4 | 0 | 0 | 0 | **7** |  |  |  |  |  |  | 0 | 0 |
| Oxacillin |  |  |  |  | 93 | 0 | 7 |  |  |  |  |  |  | 2 | 2 |
| Pirlimycin |  |  | 89 | 7 | 0 | **0** | 4 |  |  |  |  |  |  | 1 | 1 |
| Penicillin/Novobiocin |  |  |  | 100 | 0 | **0** | 0 | 0 |  |  |  |  |  | 1 | 1 |
| Tetracycline |  |  |  | 67 | 7 | 7 | **0** | 19 |  |  |  |  |  | 1 | 16 |
| Cephalothin |  |  |  |  | 96 | 4 | 0 | 0 | 0 |  |  |  |  | 2 | 2 |
| Ceftiofur |  |  | 52 | 19 | 26 | 0 | **4** |  |  |  |  |  |  | 1 | 2 |
| Sulfadimethoxine |  |  |  |  |  |  |  |  | 15 | 4 | 0 | 0 | 81 | >256 | >256 |

* Bold estimates signify isolate frequency resistant at the MIC cutoff for the respective drugs.

**Appendix 1.7** Minimum inhibitory concentration (MIC; µg/ml) distribution for *Escherichia coli* in the fall/winter season (n=5)

|  | Percent *Escherichia coli* isolates inhibited at different drug concentrations* | | | | | | | | | | | | | MIC 50 | MIC 90 |
| --- | --- | --- | --- | --- | --- | --- | --- | --- | --- | --- | --- | --- | --- | --- | --- |
| Drug concentration (µg/ml)) | ≤0.12 | 0.25 | 0.5 | 1 | 2 | 4 | 8 | 16 | 32 | 64 | 128 | 256 | >256 |  |  |
| Ampicillin | 40 | 0 | 0 | 0 | 0 | 20 | 0 | 40 |  |  |  |  |  | 8 | 16 |
| Penicillin | 20 | 0 | 0 | 0 | 20 | 0 | 0 | 60 |  |  |  |  |  | 16 | 16 |
| Erythromycin |  | 20 | 0 | 0 | 0 | 0 | 80 |  |  |  |  |  |  | 8 | 8 |
| Oxacillin |  |  |  |  | 20 | 2 | 80 |  |  |  |  |  |  | 8 | 8 |
| Pirlimycin |  |  | 20 | 0 | 0 | 0 | 80 |  |  |  |  |  |  | 8 | 8 |
| Penicillin/Novobiocin |  |  |  | 20 | 20 | 0 | 60 | 0 |  |  |  |  |  | 16 | 16 |
| Tetracycline |  |  |  | 40 | 20 | 0 | 0 | **40** |  |  |  |  |  | 2 | 16 |
| Cephalothin |  |  |  |  | 40 | 0 | 0 | **20** | 40 |  |  |  |  | 16 | 32 |
| Ceftiofur |  |  | 80 | 20 | 0 | 0 | **0** |  |  |  |  |  |  | 1 | 2 |
| Sulfadimethoxine |  |  |  |  |  |  |  |  | 20 | 20 | 0 | 0 | **60** | >256 | >256 |

* Bold estimates signify isolate frequency resistant at the MIC cutoff for the respective drugs.

**Appendix 1.8** Minimum inhibitory concentration (MIC; µg/ml) distribution for *Escherichia coli* in the spring/summer season (n=16)

|  | Percent *Escherichia coli* isolates inhibited at different drug concentrations* | | | | | | | | | | | | | MIC 50 | MIC 90 |
| --- | --- | --- | --- | --- | --- | --- | --- | --- | --- | --- | --- | --- | --- | --- | --- |
| Drug concentration (µg/ml) | ≤0.12 | 0.25 | 0.5 | 1 | 2 | 4 | 8 | 16 | 32 | 64 | 128 | 256 | >256 |  |  |
| Ampicillin | 13 | 0 | 6 | 0 | 19 | 6 | 6 | 50 |  |  |  |  |  | 8 | 16 |
| Penicillin | 13 | 0 | 0 | 6 | 0 | 0 | 6 | 75 |  |  |  |  |  | 16 | 16 |
| Erythromycin |  | 19 | 6 | 0 | 0 | 0 | 75 |  |  |  |  |  |  | 8 | 8 |
| Oxacillin |  |  |  |  | 19 | 0 | 81 |  |  |  |  |  |  | 8 | 8 |
| Pirlimycin |  |  | 13 | 0 | 0 | 0 | 88 |  |  |  |  |  |  | 8 | 8 |
| Penicillin/Novobiocin |  |  |  | 25 | 0 | 0 | 6 | 69 |  |  |  |  |  | 16 | 16 |
| Tetracycline |  |  |  | 19 | 38 | 0 | 0 | **44** |  |  |  |  |  | 2 | 16 |
| Cephalothin |  |  |  |  | 19 | 13 | 13 | **13** | 44 |  |  |  |  | 16 | 32 |
| Ceftiofur |  |  | 56 | 25 | 13 | 6 | **0** |  |  |  |  |  |  | 1 | 2 |
| Sulfadimethoxine |  |  |  |  |  |  |  |  | 6 | 0 | 13 | 6 | **75** | >256 | >256 |

* Bold estimates signify isolate frequency resistant at the MIC cutoff for the respective drugs.
