# Supplementary material for: Effect of dry cow therapy on antimicrobial resistance of mastitis pathogens post-calving
Source: Front Vet Sci. 2023 Jul 20;10:1132810. doi: 10.3389/fvets.2023.1132810 (PMC10399697; doi:10.3389/fvets.2023.1132810)
Supplement: Supplementary file 2 [file Table_2.DOCX]

Supplementary material: Univariable models for the tested antimicrobials.

| Tested drug | Variables | Levels | Coefficient | SE | P-value | 95% CI |
| --- | --- | --- | --- | --- | --- | --- |
| Penicillin | IMM infusion at dry off | No treatment | Referent | | | |
|  |  | Treatment | -0.31 | 2.180 | 0.88 | (-4.58, 3.96) |
|  | IMM infusion at dry off | No treatment | Referent | | | |
|  |  | Treatment containing penicillin | 5.64 | 2.522 | 0.02 | (0.70, 10.59) |
|  |  | Treatment other than penicillin | 1.86 | 2.127 | 0.38 | (-2.30, 6.02) |
|  | Mastitis during dry off lactation | No | Referent | | | |
|  |  | Yes | -3.19 | 2.223 | 0.15 | (-7.55, 1.16) |
|  | Season | Winter | Referent |  |  |  |
|  |  | Summer | 1.68 | 1.0776 | 0.11 | (-0.42, 3.79) |
|  | Breed | Pure breed | Referent |  |  |  |
|  |  | Mixed breed | -0.83 | 2.163 | 0.69 | (-5.07, 3.40) |
|  | Region | Southern SJV | Referent |  |  |  |
|  |  | Northern SJV | -2.93 | 2.203 | 0.18 | (-7.25, 1.38) |
|  | Parity | = 2 | Referent |  |  |  |
|  |  | > 2 | 1.56 | 1.485 | 0.29 | (-1.34, 4.47) |
|  | Dry period length | days | -0.04 | 0.053 | 0.41 | (-0.14, 0.06) |
|  | Udder hygiene score | ≤ 2 | Referent |  |  |  |
|  |  | > 2 | -1.14 | 1.863 | 0.53 | (-4.80, 2.50) |
|  | California mastitis test score at dry off | < 3 | Referent |  |  |  |
|  |  | = 3 | -1.94 | 2.65 | 0.46 | (-7.14, 3.25) |
|  | Teat end score | < 4 | Referent |  |  |  |
|  |  | 4 at any teat | 1.89 | 1.556 | 0.22 | (-1.15, 4.94) |
|  | Last DHIA SCC test | Natural log (1000cells/ml) | 0.62 | 0.503 | 0.21 | (-0.36, 1.60) |
|  |  |  |  |  |  |  |
| Ampicillin | IMM infusion at dry off | No treatment | Referent |  |  |  |
|  |  | Treatment | -0.66 | 1.462 | 0.65 | (-3.52, 2.20) |
|  | IMM infusion at dry off | No treatment | Referent |  |  |  |
|  |  | Treatment containing penicillin | 3.68 | 1.380 | <0.01 | (0.97, 6.38) |
|  |  | Treatment other than penicillin | 1.19 | 1.479 | 0.42 | (-1.70, 4.09) |
|  | Mastitis during dry off lactation | No | Referent |  |  |  |
|  |  | Yes | -1.63 | 1.376 | 0.23 | (-4.32, 1.06) |
|  | Season | Winter | Referent |  |  |  |
|  |  | Summer | 0.74 | 0.609 | 0.22 | (-0.45, 1.93) |
|  | Breed | Pure breed | Referent |  |  |  |
|  |  | Mixed breed | -0.68 | 1.051 | 0.51 | (-2.74, 1.37) |
|  | Region | Southern SJV | Referent |  |  |  |
|  |  | Northern SJV | -2.07 | 1.104 | 0.06 | (-4.23, 0.09) |
|  | Parity | = 2 | Referent |  |  |  |
|  |  | > 2 | 0.68 | 1.194 | 0.56 | (-1.65, 3.02) |
|  | Dry period length | days | -0.02 | 0.381 | 0.48 | (-0.10, 0.04) |
|  | Udder hygiene score | ≤ 2 | Referent |  |  |  |
|  |  | > 2 | -1.53 | 1.078 | 0.15 | (-3.65, 0.57) |
|  | California mastitis test score at dry off | < 3 | Referent |  |  |  |
|  |  | = 3 | -3.00 | 1.484 | 0.04 | (-5.91, -0.09) |
|  | Teat end score | < 4 | Referent |  |  |  |
|  |  | 4 at any teat | -0.37 | 0.923 | 0.68 | (-2.18, 1.43) |
|  | Last DHIA SCC test | Natural log (1000cells/ml) | 0.18 | 0.284 | 0.52 | (-0.37, 0.74) |
|  |  |  |  |  |  |  |
| Oxacillin | IMM infusion at dry off | No treatment | Referent |  |  |  |
|  |  | Treatment with IMM | 3.21 | 5.374 | 0.55 | (-7.31, 13.75) |
|  | IMM infusion at dry off | No treatment | Referent |  |  |  |
|  |  | Treatment containing oxacillin | 5.36 | 6.22 | 0.38 | (-6.82, 17.56) |
|  |  | Treatment other than oxacillin | 1.30 | 3.63 | 0.72 | (-5.82, 8.43) |
|  | Mastitis during dry off lactation | No | Referent |  |  |  |
|  |  | Yes | -13.45 | 14.055 | 0.33 | (-41.00, 14.09) |
|  | Season | Winter | Referent |  |  |  |
|  |  | Summer | 1.34 | 1.269 | 0.29 | (-1.14, 3.82) |
|  | Breed | Pure breed | Referent |  |  |  |
|  |  | Mixed breed | 0.13 | 1.216 | 0.91 | (-2.24, 2.51) |
|  | Region | Southern SJV | Referent |  |  |  |
|  |  | Northern SJV | -2.53 | 3.400 | 0.45 | (-9.20, 4.12) |
|  | Parity | = 2 | Referent |  |  |  |
|  |  | > 2 | 12.96 | 13.574 | 0.33 | (-13.63, 39.57) |
|  | Dry period length | days | -0.19 | 0.306 | 0.52 | (-0.79, 0.40) |
|  | Udder hygiene score | ≤ 2 | Referent |  |  |  |
|  |  | > 2 | -13.10 | 13.665 | 0.33 | (-39.88, 13.67) |
|  | California mastitis test score at dry off | < 3 | Referent |  |  |  |
|  |  | = 3 | 2.76 | 0.743 | <0.01 | (1.30, 4.22) |
|  | Teat end score | < 4 | Referent |  |  |  |
|  |  | 4 at any teat | -13.02 | 13.699 | 0.34 | (-39.87, 13,82) |
|  | Last DHIA SCC test | Natural log (1000cells/ml) | -0.07 | 0.386 | 0.84 | (-0.83, 0.68) |
|  |  |  |  |  |  |  |
| Ceftiofur | IMM infusion at dry off | No treatment | Referent |  |  |  |
|  |  | Treatment with IMM | 0.27 | 0.143 | 0.05 | (-0.005, 0.55) |
|  | IMM infusion at dry off | No treatment | Referent |  |  |  |
|  |  | Treatment containing ceftiofur | 0.19 | 0.155 | 0.21 | (-0.11, 0.49) |
|  |  | Treatment other than ceftiofur | 0.34 | 0.206 | 0.10 | (-0.06, 0.74) |
|  | Mastitis during dry off lactation | No | Referent |  |  |  |
|  |  | Yes | -0.11 | 0.181 | 0.51 | (-0.47, 0.23) |
|  | Season | Winter | Referent |  |  |  |
|  |  | Summer | 0.43 | 0.164 | <0.01 | (0.11, 0.75) |
|  | Breed | Pure breed | Referent |  |  |  |
|  |  | Mixed breed | 0.002 | 0.117 | 0.98 | (-0.22, 0.23) |
|  | Region | Southern SJV | Referent |  |  |  |
|  |  | Northern SJV | -0.27 | 0.130 | 0.03 | (-0.53, -0.02) |
|  | Parity | = 2 | Referent |  |  |  |
|  |  | > 2 | 0.35 | 0.180 | 0.04 | (0.003, 0.70) |
|  | Dry period length | days | -0.01 | 0.003 | <0.01 | (-0.02, -0.01) |
|  | Udder hygiene score | ≤ 2 | Referent |  |  |  |
|  |  | > 2 | 0.82 | 0.379 | 0.02 | (0.08, 1.57) |
|  | California mastitis test score at dry off | < 3 | Referent |  |  |  |
|  |  | = 3 | -0.03 | 0.196 | 0.85 | (-0.42, 0.34) |
|  | Teat end score | < 4 | Referent |  |  |  |
|  |  | 4 at any teat | 1.24 | 0.731 | 0.08 | (-0.19, 2.67) |
|  | Last DHIA SCC test | Natural log (1000cells/ml) | 0.16 | 0.035 | <0.01 | (0.09, 0.23) |
| Cephalothin | IMM infusion at dry off | No treatment | Referent |  |  |  |
|  |  | Treatment with IMM | 7.05 | 1.079 | <0.01 | (4.93, 9.16) |
|  | IMM infusion at dry off | No treatment | Referent |  |  |  |
|  |  | Treatment containing ceftiofur | 6.43 | 0.639 | <0.01 | (5.17, 7.68) |
|  |  | Treatment other than ceftiofur | 7.10 | 1.031 | <0.01 | (5.08, 9.12) |
|  | Mastitis during dry off lactation | No | Referent |  |  |  |
|  |  | Yes | -8.28 | 1.272 | <0.01 | (-10.78, -5.79) |
|  | Season | Winter | Referent |  |  |  |
|  |  | Summer | 7.31 | 1.187 | <0.01 | (4.98, 9.64) |
|  | Breed | Pure breed | Referent |  |  |  |
|  |  | Mixed breed | 0.96 | 0.593 | 0.10 | (-0.19, 2.13) |
|  | Region | Southern SJV | Referent |  |  |  |
|  |  | Northern SJV | -0.61 | 0.582 | 0.29 | (-1.75, 0.53) |
|  | Parity | = 2 | Referent |  |  |  |
|  |  | > 2 | 7.07 | 1.142 | <0.01 | (4.83, 9.31) |
|  | Dry period length | days | -0.08 | 0.042 | 0.04 | (-0.16, -0.0009) |
|  | Udder hygiene score | ≤ 2 | Referent |  |  |  |
|  |  | > 2 | -7.43 | 1.143 | <0.01 | (-9.67, -5.19) |
|  | California mastitis test score at dry off | < 3 | Referent |  |  |  |
|  |  | = 3 | -7.45 | 1.161 | <0.01 | (-9.72, -5.17) |
|  | Teat end score | < 4 | Referent |  |  |  |
|  |  | 4 at any teat | -6.94 | 1.080 | <0.01 | (-9.05, -4.82) |
|  | Last DHIA SCC test | Natural log (1000cells/ml) | 0.15 | 0.312 | 0.62 | (-0.45, 0.76) |
|  |  |  |  |  |  |  |
| Erythromycin | IMM infusion at dry off | No treatment | Referent |  |  |  |
|  |  | Treatment with IMM | -0.001 | 0.414 | 0.99 | (-0.81, 0.81) |
|  | Mastitis during dry off lactation | No | Referent |  |  |  |
|  |  | Yes | -0.52 | 0.221 | 0.01 | (-0.95, -0.08) |
|  | Season | Winter | Referent |  |  |  |
|  |  | Summer | -0.37 | 0.251 | 0.13 | (-0.87, 0.11) |
|  | Breed | Pure breed | Referent |  |  |  |
|  |  | Mixed breed | -0.71 | 0.196 | <0.01 | (-1.09, -0.32) |
|  | Region | Southern SJV | Referent |  |  |  |
|  |  | Northern SJV | -1.04 | 0.187 | <0.01 | (-1.41, -0.67) |
|  | Parity | = 2 | Referent |  |  |  |
|  |  | > 2 | 0.51 | 0.182 | <0.01 | (0.15, 0.86) |
|  | Dry period length | days | -0.01 | 0.003 | <0.01 | (-0.02, -0.005) |
|  | Udder hygiene score | ≤ 2 | Referent |  |  |  |
|  |  | > 2 | -1.02 | 0.494 | 0.03 | (-1.98, -0.05) |
|  | California mastitis test score at dry off | < 3 | Referent |  |  |  |
|  |  | = 3 | -1.06 | 0.445 | 0.01 | (-1.94, -0.19) |
|  | Teat end score | < 4 | Referent |  |  |  |
|  |  | 4 at any teat | -0.73 | 0.213 | <0.01 | (-1.15, -0.32 |
|  | Last DHIA SCC test | Natural log (1000cells/ml) | 0.02 | 0.070 | 0.75 | (-0.11, 0.15) |
|  |  |  |  |  |  |  |
| Pirlimycin | IMM infusion at dry off | No treatment | Referent |  |  |  |
|  |  | Treatment with IMM | 0.88 | 1.228 | 0.47 | (-1.52, 3.29) |
|  | Mastitis during dry off lactation | No | Referent |  |  |  |
|  |  | Yes | -10.73 | 2.665 | <0.01 | (15.96, -5.51) |
|  | Season | Winter | Referent |  |  |  |
|  |  | Summer | 1.13 | 0.942 | 0.23 | (-0.71, 2.97) |
|  | Breed | Pure breed | Referent |  |  |  |
|  |  | Mixed breed | -10.63 | 2.640 | <0.01 | (-15.80, -5.45) |
|  | Region | Southern SJV | Referent |  |  |  |
|  |  | Northern SJV | -1.41 | 1.056 | 0.18 | (-3.48, 0.65) |
|  | Parity | = 2 | Referent |  |  |  |
|  |  | > 2 | 3.51 | 1.670 | 0.03 | (0.24, 6.79) |
|  | Dry period length | days | -0.04 | 0.084 | 0.59 | (-0.21, 0.12) |
|  | Udder hygiene score | ≤ 2 | Referent |  |  |  |
|  |  | > 2 | 0.80 | 0.542 | 0.14 | (-0.26, 1.86) |
|  | California mastitis test score at dry off | < 3 | Referent |  |  |  |
|  |  | = 3 | 0.28 | 2.208 | 0.89 | (-4.04, 4.61) |
|  | Teat end score | < 4 | Referent |  |  |  |
|  |  | 4 at any teat | 2.94 | 1.143 | 0.01 | (0.70, 5.19) |
|  | Last DHIA SCC test | Natural log (1000cells/ml) | -0.14 | 0.357 | 0.67 | (-0.84, 0.55) |
|  |  |  |  |  |  |  |
| Tetracycline | IMM infusion at dry off | No treatment | Referent |  |  |  |
|  |  | Treatment with IMM | 3.22 | 0.398 | <0.01 | (2.44, 4.01) |
|  | Mastitis during dry off lactation | No | Referent |  |  |  |
|  |  | Yes | -11.88 | 2.67 | <0.01 | (-17.12, -6.65) |
|  | Season | Winter | Referent |  |  |  |
|  |  | Summer | 2.27 | 2.947 | 0.44 | (-3.50, 8.04) |
|  | Breed | Pure breed | Referent |  |  |  |
|  |  | Mixed breed | -12.24 | 2.735 | <0.01 | (-17.60, -6.88) |
|  | Region | Southern SJV | Referent |  |  |  |
|  |  | Northern SJV | -2.52 | 2.684 | 0.34 | (-7.78, 2.73) |
|  | Parity | = 2 | Referent |  |  |  |
|  |  | > 2 | 11.54 | 2.513 | <0.01 | (6.61, 16.46) |
|  | Dry period length | days | -0.14 | 0.046 | <0.01 | (-0.23, -0.05) |
|  | Udder hygiene score | ≤ 2 | Referent |  |  |  |
|  |  | > 2 | 2.87 | 1.64 | 0.08 | (-0.35, 6.09) |
|  | California mastitis test score at dry off | < 3 | Referent |  |  |  |
|  |  | = 3 | -13.03 | 2.91 | <0.01 | (-18.74, -7.32) |
|  | Teat end score | < 4 | Referent |  |  |  |
|  |  | 4 at any teat | 5.67 | 4.16 | 0.17 | (-2.48, 13.83) |
|  | Last DHIA SCC test | Natural log (1000cells/ml) | 1.65 | 0.478 | <0.01 | (0.71, 2.59) |
